# Retinoic Acid and GM-CSF Coordinately Induce Retinal Dehydrogenase 2 (RALDH2) Expression through Cooperation between the RAR/RXR Complex and Sp1 in Dendritic Cells

**DOI:** 10.1371/journal.pone.0096512

**Published:** 2014-05-02

**Authors:** Yoshiharu Ohoka, Aya Yokota-Nakatsuma, Naoko Maeda, Hajime Takeuchi, Makoto Iwata

**Affiliations:** 1 Laboratory of Immunology, Kagawa School of Pharmaceutical Sciences, Tokushima Bunri University, Sanuki-shi, Kagawa, Japan; 2 Japan Science and Technology Agency, CREST, Chiyoda-ku, Tokyo, Japan; Deakin School of Medicine, Australia

## Abstract

Retinoic acid (RA)-producing dendritic cells (DCs) play critical roles in gut immunity. Retinal dehydrogenase 2 (RALDH2) encoded by *Aldh1a2* is a key enzyme for generating RA in DCs. Granulocyte–macrophage colony-stimulating factor (GM-CSF) potently induces RALDH2 expression in DCs in an RA-dependent manner, and RA alone weakly induces the expression. However, how GM-CSF and RA induce RALDH2 expression remains unclear. Here, we show that GM-CSF-induced activation of the transcription factor Sp1 and RA-dependent signaling via the RA receptor (RAR)/retinoid X receptor (RXR) complex contribute to *Aldh1a2* expression. The RAR antagonist LE540 and the Sp1 inhibitor mithramycin A inhibited GM-CSF-induced *Aldh1a2* expression in fms-related tyrosine kinase 3 ligand-generated bone marrow-derived DCs (BM-DCs). ERK and p38 MAPK inhibitors suppressed GM-CSF-induced nuclear translocation of Sp1 and *Aldh1a2* expression. Sp1 and the RARα/RXRα complex bound to GC-rich Sp1-binding sites and an RA response element (RARE) half-site, respectively, near the TATA box in the mouse *Aldh1a2* promoter. The DNA sequences around these sites were highly conserved among different species. In the presence of RA, ectopic expression of RARα/RXRα and Sp1 synergistically enhanced *Aldh1a2* promoter-reporter activity. GM-CSF did not significantly induce *Aldh1a2* expression in plasmacytoid DCs, peritoneal macrophages, or T cells, and the *Aldh1a2* promoter in these cells was mostly unmethylated. These results suggest that GM-CSF/RA-induced RALDH2 expression in DCs requires cooperative binding of Sp1 and the RAR/RXR complex to the *Aldh1a2* promoter, and can be regulated by a DNA methylation-independent mechanism.

## Introduction

Dendritic cells (DCs) in gut-related lymphoid organs, mesenteric lymph nodes (MLNs) and Peyer's patches, produce the vitamin A metabolite retinoic acid (RA), and thereby imprint gut-homing specificity on lymphocytes by inducing or enhancing the expression of the gut-homing receptors, integrin α4β7 and the chemokine receptor CCR9 [1]. RA also modulates the differentiation of naïve CD4^+^ T cells to become Th1, Th2, Th17, or Foxp3^+^ inducible regulatory T cells [2]–[9]. Because an RA receptor (RAR) α isoform deficiency limits fundamental T cell signaling [10], basal levels of RA may be essential for T-cell activation and the subsequent development of effector T cells.

DCs in MLNs, Peyer's patches, and the lamina propria (LP) of the small intestine express the RA-producing enzyme retinal dehydrogenase 2 (RALDH2) encoded by *Aldh1a2* [1], [8], [11]. High RALDH2 activity levels have been found in a CD103^+^ mature conventional DC (cDC) subset of MLN-DCs [11], [12]. However, the molecular mechanism underlying the induction of RALDH2 expression remains unclear.

Multiple factors, including RA, granulocyte–macrophage colony-stimulating factor (GM-CSF), interleukin (IL)-4, IL-13, Toll-like receptor (TLR) ligands, peroxisome proliferator-activated receptor-γ agonists, and β-catenin-activating factors can participate in inducing *Aldh1a2* expression in DCs [11], [13]–[19]. GM-CSF is one of the most potent inducers of *Aldh1a2* expression in DCs, and it appears to play an important role in the steady-state expression of RALDH2 in MLN-DCs [11], although its contribution can be exerted by other factors depending on the rearing conditions or the animal strains used [20]. IL-4 is also a potent inducer of *Aldh1a2* expression in DCs, and GM-CSF and IL-4 synergistically enhance RALDH2 expression, although IL-4 is not essential for the steady-state expression of RALDH2 in MLN-DCs [11]. TLR stimulation alone induces low RALDH2 expression levels in immature DCs; however, it markedly enhances GM-CSF-induced *Aldh1a2* expression and maturation [11]. However, the involvement of TLR stimulation in *Aldh1a2* expression in gut DCs in vivo remains controversial, as different groups have reported conflicting results [12], [19], [20].

There may be redundant pathways for inducing or enhancing *Aldh1a2* expression, and alternative pathways may be utilized under certain circumstances, particularly in gene-knockout mice. However, RA and β-catenin do appear to be essential for *Aldh1a2* expression in DCs, because a deficiency in vitamin A or β-catenin almost completely inhibits *Aldh1a2* expression and RALDH2 activity in DCs [11], [16].

In the present study, we assessed the molecular mechanisms involved in GM-CSF-induced and RAR-dependent *Aldh1a2* expression in DCs. RA alone induces weak RALDH2 expression in fms-related tyrosine kinase 3 ligand (Flt3L)-generated bone marrow (BM)-derived immature DCs (BM-DCs); however, it is required for GM-CSF-induced RALDH2 expression in these cells [11]. We found that the RAR/retinoid X receptor (RXR) complex bound to an RA response element (RARE) half-site located near the TATA box in the mouse *Aldh1a2* promoter. This promoter was located within a CpG island, and contained multiple Sp1 binding sites, including one that was near the RARE half-site. Thus, we propose that *Aldh1a2* expression in normal DCs requires GM-CSF/RA-dependent activation of the *Aldh1a2* promoter through the cooperative binding of Sp1 and RARα/RXRα to this promoter, and is regulated by a DNA methylation-independent mechanism.

## Materials and Methods

### Ethics statement

All animal experiments were performed according to the protocols approved by the Animal Care and Use Committee of Tokushima Bunri University (Approved Number: KP13-041-001).

### Mice

B10.D2 mice and C57BL/6 mice were from Japan SLC and CLEA Japan, respectively.

### Reagents

All-*trans*-RA, lipopolysaccharide (LPS), cycloheximide, mithramycin A, and phorbol-12-myristate-13-acetate (PMA) were purchased from Sigma-Aldrich. PD98059 and SB203580 were purchased from Merck-Calbiochem. LE540 was gifts from Dr. H. Kagechika (Tokyo Medical and Dental University). Mouse GM-CSF and CpG-ODN1826 were purchased from PeproTech and InvivoGen, respectively.

### DC culture

To obtain BM-DCs, BM progenitors were harvested from femurs and tibias of B10.D2 or C57BL/6 mice, and were immunomagnetically sorted by negative selection using the EasySep Mouse Hematopoietic Progenitor Cell Enrichment Kit (StemCell Technologies). BM-DCs were generated by culturing them for 8 days in complete medium (DMEM supplemented with 10% heat-inactivated fetal bovine serum (Equitech-Bio), 2 mM L-glutamine, 1 mM sodium pyruvate, 1 × MEM non-essential amino acids, 50 µM 2-mercaptoethanol, 20 mM HEPES-NaOH (pH 7.2), 100 U/ml penicillin and 100 µg/ml streptomycin) containing 20% culture supernatant of a mouse Flt3L-transfected CHO cell line. In some experiments, Flt3L-generated BM-DCs were stained with allophycocyanin-labeled anti-CD11c antibody (Ab) (eBioscience) and phycoerythrin-labeled anti-B220 Ab (BD Biosciences). CD11c^+^B220^+^ and CD11c^+^B220^−^ cells were purified by FACS sorting with a FACSAria cell sorter (BD Biosciences) and used as plasmacytoid DCs (pDCs) and cDCs, respectively. In some experiments, the cellular RALDH2 (ALDH1A2) protein was analyzed by Western blotting as previously described [11].

### Real-time PCR

Total RNA was isolated from cells using RNeasy Mini Kit, and cDNA was generated using QuantiTect Reverse Transcription Kit (both from Qiagen), according to the manufacturer's instructions. The level of *Aldh1a2* gene expression was determined by real-time PCR in triplicates with Power SYBR Green PCR Master Mix (Applied Biosystems) and gene-specific primers (Table S1) using an Applied Biosystems 7500 or 7900 Real-time PCR system. Quantitative normalization of cDNA in each sample was obtained by the Δ*C*T method (*Aldh1a2 C*T – *Rplp0 C*T).

### Construction of plasmids

For the analysis of the *Aldh1a2* promoter, the 5′-flanking region of the mouse *Aldh1a2* gene was cloned by PCR using mouse genomic DNA as a template and specific reverse and forward primers (Table S1). A 2.8-kb PCR product (−2,600 to +182) was inserted into the pGEM-T Easy vector (Promega), according to the manufacturer's instructions. The insert sequence of the resulting vector was identical to the sequence found in NCBI, GenBank™ accession number NT_039474.8. The insert was then ligated in the promoterless firefly luciferase reporter vectors, pGL3-basic and pGL4-basic (Promega), at the *Kpn*I and *Xho*I sites. Various truncated forms of the mouse *Aldh1a2* promoter were also created by PCR using reverse and forward primers (Table S1). The products were inserted into the pGL3-basic vector at the *Kpn*I and *Hind*III sites. Site-directed mutagenesis of putative RARE half-sites in the *Aldh1a2* promoter was introduced by the QuickChange Site-directed Mutagenesis Kit (Agilent Technologies), using reverse and forward primers (Table S1), according to the manufacturer's instructions. Various promoter vectors lacking a GC-rich region were also created by the QuickChange Site-directed Mutagenesis Kit, using reverse and forward primers (Table S1). In some experiments, PCR fragments amplified from pGL3-RALDH2 using reverse and forward primers (Table S1) were inserted into the *Spe*I and *Hind*III sites in the CpG dinucleotide-free pCpGL luciferase vector, a kind gift from Dr. M. Rehli (University Hospital of Regensburg, Germany) [21], and GT115 *E. coli* cells (InvivoGen) were transformed with the plasmids. The mouse *Sp1* was cloned by PCR using mouse spleen (SPL) cDNA as a template and specific reverse and forward primers (Table S1). Then the PCR product was inserted into the pGEM-T Easy vector, according to the manufacturer's instructions. The PCR product was then inserted into pCMV-Myc expression vector using *Eco*RI and *Sal*I sites to obtain the Sp1 expression vector pCMV-Myc-Sp1. The expression vector containing only the DNA-binding domain of Sp1 (pCMV-Myc-Sp1db) was also constructed by PCR using specific reverse and forward primers (Table S1). All the constructs were verified by sequencing using Big Dye Terminator (Applied Biosystems) on an ABI 3130 sequencer (Applied Biosystems). pSG5-RARα was a kind gift from Dr. P. Chambon (IGBMC, Université Louis Pasteur) [22]. pSG5-RXRα was previously described [23].

### Subcellular fractionation

Cytosolic and nuclear protein extracts from BM-DCs were prepared by means of NE-PER Nuclear and Cytoplasmic Extraction Reagent (Thermo Scientific) according to the manufacturer's instructions. The purity of the cytosolic fraction and nuclear fraction was verified using anti-α-tubulin (Sigma-Aldrich) and anti-lamin B1 (MBL) Abs, respectively.

### DNA affinity precipitation (DNAP) assay

DNAP assay was performed as previously described [23]. The biotin-labeled DNA probes (Sigma-Aldrich) were annealed to complementary oligonucleotides. COS-7 cells were transfected with 1 µg of expression vectors using LipofectAmine 2000 (Invitrogen), according to the manufacturer's instructions. Transfected COS-7 cells or nuclear fractions of BM-DCs were lysed or diluted with DNAP binding buffer [25 mM Tris-HCl (pH 8.0), 100 mM NaCl, 1 mM EDTA, 0.25% NP-40, 1 mM DTT and Complete Protease Inhibitor Cocktail (Nacalai Tesque)], respectively. Cell debris was removed by centrifugation (20,000×*g*) for 10 min. Lysates were first incubated with Streptavidin-Sepharose beads (GE Healthcare) for 30 min to eliminate nonspecific binding and then incubated with 1.5 µg of poly(dI-dC) and 2 µg of biotinylated DNA probe for 1 h at 4°C. Streptavidin-Sepharose beads were then added and incubated with these mixtures for an additional 30 min at 4°C. After washing the beads three times in DNAP binding buffer, precipitated proteins were eluted in SDS-PAGE sample buffer. Samples were analyzed SDS-PAGE followed by Western blot analysis using anti-Sp1 (Santa Cruz Biotechnology), anti-Myc (Nacalai Tesque), anti-RARα (Santa Cruz Biotechnology), and anti-RXRα (Santa Cruz Biotechnology) Abs.

### Transfection and luciferase assay

COS-7 cells were maintained in complete DMEM medium. Cells were seeded into 12- or 6-well plates (5×10^5^ or 1×10^6^ cells/well, respectively) and transfected with 1.25 µg of various reporter vectors, 0.5–1.5 µg of expression vectors, and 0.025 µg of pRL-TK (Promega) unless otherwise indicated using LipofectAmine 2000, according to the manufacturer's instructions. After 24 h, the cells were transferred into new 48-well plates, and then stimulated with 5 ng/ml PMA and/or 100 nM RA. Sixteen hours after stimulation, the cells were washed in PBS, and lysed in 1 × passive lysis buffer (Promega). The firefly and Renilla luciferase activities were measured by the dual luciferase assay system (Promega) in a luminometer (Turner TD-20/20), according to the manufacturer's instructions. All experiments were carried out in triplicates, and the firefly luciferase activity was normalized by the Renilla luciferase activity.

### Chromatin Immunoprecipitation (ChIP) Assay

ChIP assay was performed as previously described [23]. Briefly, aliquots of cultured BM-DCs (2×10^6^) were fixed with 1% formaldehyde at 37°C for 10 min. Cross-linking reactions were quenched with 150 mM glycine. Cells were washed, suspended in SDS lysis buffer, and sonicated to shear the chromatin into 200–500 bp fragments using a sonicator (Bioruptor UCW-201). After centrifugation to remove debris, aliquots were incubated with 5 µg anti-Sp1 or anti-RARα Ab or control IgG1 overnight, followed by incubation with protein G beads (Cell Signaling Technology) for 1 h at 4°C with rotation. The immunoprecipitates were sequentially washed with ChIP dilution buffer, high-salt buffer, LiCl buffer, and TE buffer. The DNA-protein complex was eluted in SDS lysis buffer, and was de-crosslinked. After proteins were digested with proteinase K, DNA was isolated and subjected to PCR analysis. An aliquot of chromatin that was not incubated with an antibody was used as the input DNA control. The binding of Sp1 or RARα to the *Aldh1a2* promoter site was estimated by real-time PCR with Power SYBR Green PCR Master Mix and gene-specific primers (Table S1). The binding levels were expressed as % of input DNA and was calculated from Δ*C*T (Δ*C*T  =  input *C*T – ChIP *C*T) according to the following equation: % total  =  2^Δ*C*T^.

### Analysis of DNA methylation with a bisulfite method

Genomic DNA was prepared using the DNA fast pure kit (TAKARA). DNA was denatured, modified with sodium bisulfite, purified, desulfonated using a MethylEasy Xceed kit (Human Genetic Signatures), according to the manufacturer's instructions, and applied to nested PCR for bisulfite sequencing. The primers for the first PCR and the nested PCR were shown in Table S1. PCR products were inserted into the pGEM-T Easy vector, and 10–20 clones from each sample were sequenced using Big Dye Terminator on an ABI 3130 sequencer. The methylation state for each CpG site in the amplicon sequences was analyzed by using a web-based freely available quantification tool for methylation analysis (QUMA; http://quma.cdb.riken.jp/top/index_j.html) [24]. The sequences of the 5′-flanking regions of the mouse *Aldh1a2* gene, the human *ALDH1A2* gene (GenBank™ accession number NT_010194.17), the cattle ALDH1A2 gene (NW_003104280.1), the rat Aldh1a2 gene (NW_003809872.1), the chicken ALDH1A2 gene (NW_003763854.1), and the zebrafish aldh1a2 gene (NW_003039680.2) were obtained from NCBI Genome Resources.

### Methylation of vectors and oligonucleotides

The pCpGL vector containing a 0.53-kb fragment (−373 to +182) of the *Aldh1a2* promoter and the double-stranded oligonucleotides for DNAP assay were methylated with CpG methyltransferase (M.SssI) and S-adenosylmethionine (New England Biolabs), according to the manufacturer's instructions. Mock methylation reactions did not contain M.SssI. Methylated and mock methylated vectors and oligonucleotides were purified by phenol-chloroform extraction and ethanol precipitation [25].

### Analysis of transcription factor binding sites and CpG islands

The genomic DNA sequence of the *Aldh1a2* gene was analyzed using the MatInspector computer program (http://www.genpmatix.de/onlone-help/help-ma-matinspector/matinspector-help.html) or TFSEARCH (http://www.cbrc.jp/research/db/TFSEARCH.html) to find specific sequences, including some transcription factor-binding sites. CpG islands were searched using the NCBI MapViewer (http://www.ncbi.nlm.nih.gov/mapview/) analysis tool and the CpG island Searcher (http://www.uscnorris.com/cpgislands2).

### Statistical analysis

Statistical comparisons were carried out by using the two-tailed unpaired Student's *t*-test. The *p* value < 0.05 was considered significant.

## Results

### The transcription factor Sp1 participates in inducing *Aldh1a2* expression

**Figure 1:**
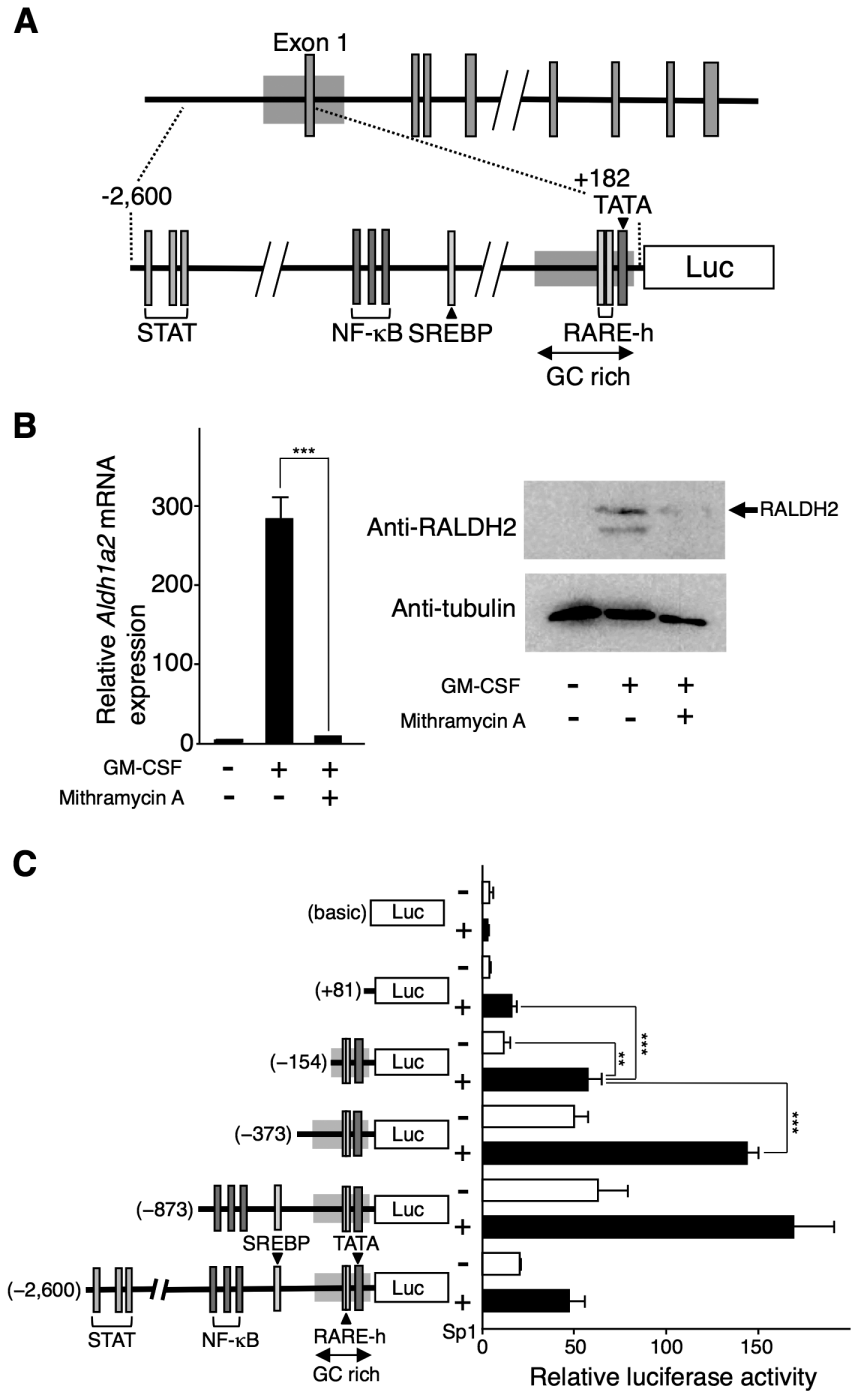
Sp1 participates in the *Aldh1a2* expression. (**A**) The genomic organization of the mouse *Aldh1a2* gene and its 5′-flanking region is shown. A fragment containing exon 1 and its 5′-flanking region from −2,600 to +182 was inserted into reporter vectors. DNA binding sites (STAT-binding sites, NF-κB binding sites, a SREBP binding site, and putative RARE half-sites (RARE-h)), the TATA box, and the GC-rich region in the fragment are indicated. (**B**) Flt3L-generated BM-DCs were cultured with 10 ng/ml GM-CSF for 16 h in the presence or absence of 1 µM mithramycin A. After the culture, *Aldh1a2* mRNA expression was assessed by real-time PCR (*Left panel*), and protein expression of RALDH2 (ALDH1A2) and α-tubulin was analyzed by Western blotting (*Right panel*). Relative mRNA expression levels were calculated by defining the *Aldh1a2* mRNA expression level in the cells incubated with medium alone for 16 h was set to 1 (*Left panel*). Data are representative of three (*Left panel*) or two (*Right panel*) independent experiments. (**C**) Serial-deletion fragments derived from the 5′-flanking region of the mouse *Aldh1a2* gene were inserted in the reporter vector, pGL3-basic. COS-7 cells were transfected in triplicate with one of the deletion constructs (1.25 µg) or the pGL3-RALDH2 (−2,600) reporter vector (1.25 µg) in combination with or without the 0.5 µg of pCMV-Myc-Sp1 expression vector. One day after the transfection, luciferase activity was measured. Relative promoter activities were calculated by arbitrarily defining the activity of pGL3-basic alone as 1. Data are presented as mean + SD of triplicate cultures. Statistical significance between two groups was determined by the Student's *t* test (**p<0.01, ***p<0.001). Data are representative of three independent experiments.

Mouse *Aldh1a2* that encodes RALDH2 comprises 14 exons that are divided by 13 introns spanning more than 50 kb of genomic DNA, and it is localized on chromosome 9 [26], [27] (Figure S1A). Several CpG islands were found in *Aldh1a2* and its upstream region using NCBI MapViewer. We analyzed a 7,000-bp fragment containing TSS and the first exon with the CpG island searcher, and identified a 1,445-bp (−816 to +629) region as a CpG island (Figure S1B). The 5′-flanking region of the first exon included typical mammalian promoter consensus elements, a TATA box (−38), GC-rich regions, a sterol regulatory element-binding protein (SREBP)-binding region (−417), and NF-κB-binding sites (−647, −622, and −575) in accord with a previous report [27]. In this CpG island, there were no typical RARE sites, although we found two putative RARE half-sites (−75 and −49) near the TATA box (Figure 1A). Putative STAT-binding sites (−2217, −2172, and −2153) were also found far upstream from these sites.

The transcription factor Sp1 is known to bind to GC-rich promoter regions [28]. We found that mithramycin A, which inhibits Sp1 binding to GC-rich regions [29], inhibited GM-CSF-induced *Aldh1a2* mRNA expression and RALDH2 protein expression in Flt3L-generated BM-DCs (Figure 1B). However, it markedly reduced BM-DC viability only in the absence of GM-CSF (data not shown).

To assess the effect of Sp1 on *Aldh1a2* promoter activity, we searched for a suitable cell line to construct a reporter assay system. We first tested several DC-like and macrophage-like cell lines for their capability to express *Aldh1a2* or their utility for ectopic gene expression. None of these cell lines allowed sufficient transfection or provided signals from the transfectants for our analysis. Finally, we chose irrelevant COS-7, a fibroblast-like cell line derived from African Green Monkey kidney cells, and transfected these cells with an Sp1 expression vector and a pGL3 reporter vector that included a 2.8-kb fragment (−2,600 to +182) of the 5′-flanking region of *Aldh1a2* [pGL3-RALDH2 (−2,600)] (Figure 1A). *Aldh1a2* promoter activity was enhanced by the ectopic expression of Sp1 in a dose-dependent manner (Figure 1C and Figure S2). A 1,727-bp deletion (−2,600 to −872) in the 5′-flanking region of this gene significantly enhanced Sp1-induced *Aldh1a2* promoter activity (Figure 1C). There may have been a negative regulatory region in this deleted fragment. Deleting the next 500 bp (−873 to −372) slightly reduced *Aldh1a2* promoter activity. Although further deletion significantly reduced this activity, a short construct that contained a 0.31-kb fragment (−154 to +156) still showed significant responsiveness to Sp1. These results suggest that the 5′-flanking region up to −373 is nearly sufficient for Sp1-dependent induction of *Aldh1a2* promoter activity.

### MAPK activation is required for GM-CSF-induced *Aldh1a2* expression and Sp1 nuclear translocation

**Figure 2:**
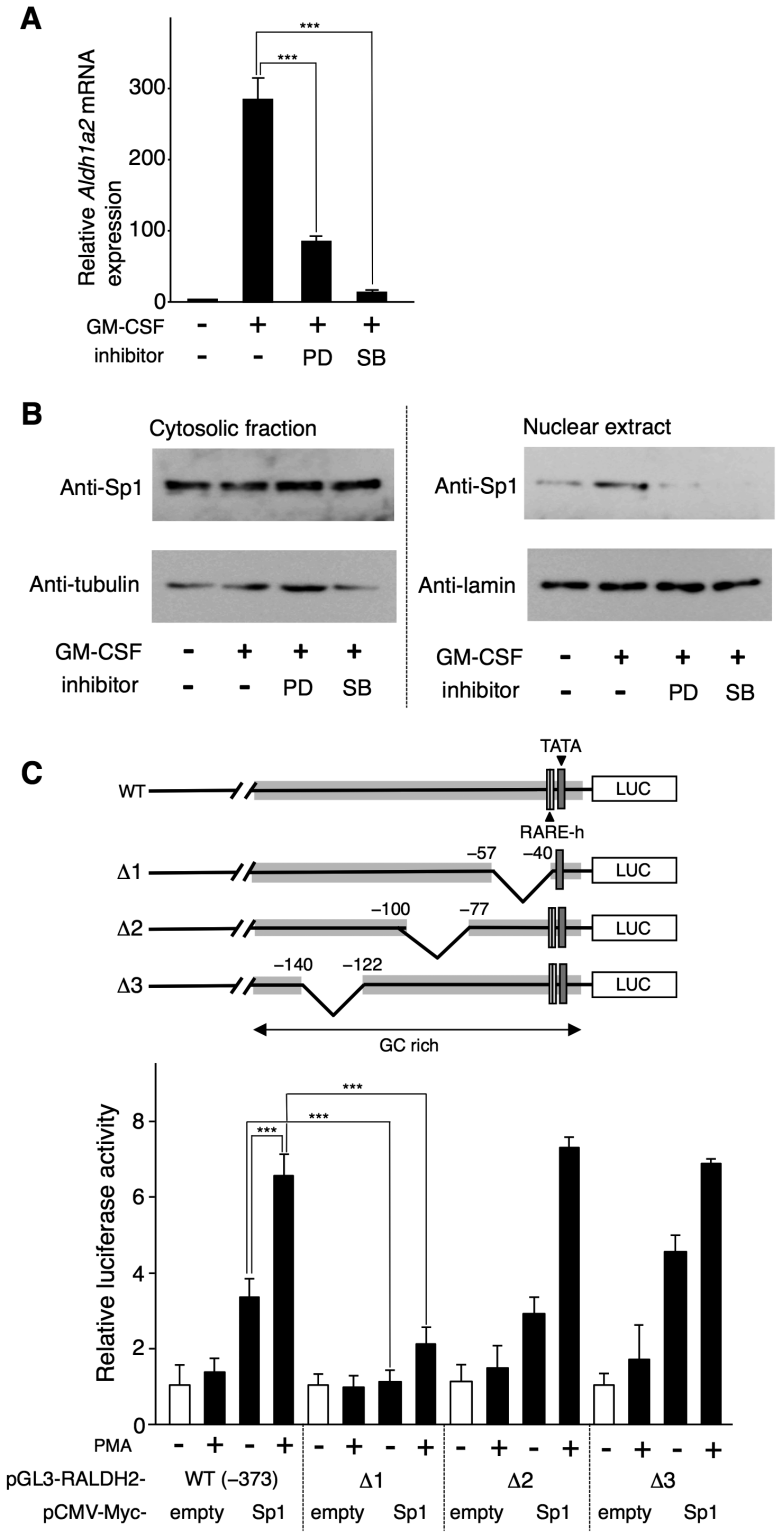
The MEK1-ERK-signaling pathway and the p38 MAPK-signaling pathway are required for the GM-CSF-induced *Aldh1a2* expression and nuclear translocation of Sp1 in BM-DCs. (**A**) Flt3L-generated BM-DCs were cultured with or without 10 ng/ml GM-CSF for 16 h in the presence or absence of 50 µM PD98059 (PD) or 25 µM SB203580 (SB). After the culture, *Aldh1a2* gene expression was assessed by real-time PCR. The *Aldh1a2* mRNA expression level in the cells incubated with medium alone for 16 h was set to 1. The results are shown as the mean + SD of triplicate cultures. Statistical significance was determined by the Student's *t* test (***p<0.001). (**B**) BM-DCs were cultured with or without 10 ng/ml GM-CSF in the presence or absence of 50 µM PD98059 or 25 µM SB203580 for 16 h. After the culture, cytosolic and nuclear proteins were analyzed for the presence of Sp1, α-tubulin, and lamin B1 by Western blotting. (**C**) Structures of the deletion constructs used in this figure are shown. Δ1, Δ2, and Δ3 constructs were prepared by deleting the region spanning from the −56 to −41, −99 to −78, and −139 to −123, respectively, from the pGL3-RALDH2 (−373) reporter vector. COS-7 cells were transfected in triplicate with the 1.25 µg of pGL3-RALDH2 (−373) reporter vector or the deletion mutants in combination with 0.5 µg of the pCMV-Myc-Sp1 expression vector or control empty vector. One day after the transfection, cells were stimulated with or without 5 ng/ml PMA, and luciferase activity was measured. Relative promoter activities were calculated by arbitrarily defining the activity of pGL3-basic alone as 1. Data are presented as mean + SD of triplicate cultures. Statistical significance between two groups was determined by the Student's *t* test (**p<0.01, ***p<0.001; NS, not significant). Data are representative of three independent experiments.

It is known that optimal *Aldh1a2* expression in CD103^+^ MLN-DCs depends on either the MEK1/2-ERK pathway or the p38α MAPK pathway [17], [30], and that GM-CSF induces the activation of MAPKs, including ERK and p38 MAPK [31]. Accordingly, the GM-CSF-induced *Aldh1a2* expression in BM-DCs was inhibited by the MEK1 inhibitor PD98059 and the p38 MAPK inhibitor SB203580 (Figure 2A). It is also known that ERK and p38 MAPK activation contributes to Sp1 nuclear translocation [32], [33]. Indeed, we found that Sp1 nuclear localization was enhanced by GM-CSF stimulation and was inhibited by treating BM-DCs with PD98059 and SB203580 (Figure 2B). These results suggest that ERK and p38 MAPK activation is essential for GM-CSF-induced Sp1 nuclear translocation and *Aldh1a2* expression in BM-DCs.

To activate ERK and MAPK in our reporter assay system, we added PMA to a culture of COS-7 cells that were transfected with the reporter vector that contained the 0.53-kb fragment (−373 to +156) of the 5′-flanking region of *Aldh1a2* [pGL3-RALDH2 (−373)] and an Sp1 expression vector. PMA treatment enhanced Sp1-induced *Aldh1a2* promoter activity (Figure 2C). Deleting a 16-bp (−56 to −41) region, but not a 22-bp (−99 to −78) or 17-bp (−139 to −123) region, resulted in a dramatic reduction in Sp1-induced reporter activity in the presence or absence of PMA. The results suggest that the short GC-rich region near the TATA box is essential for Sp1- and MAPK-dependent *Aldh1a2* expression.

**Figure 3:**
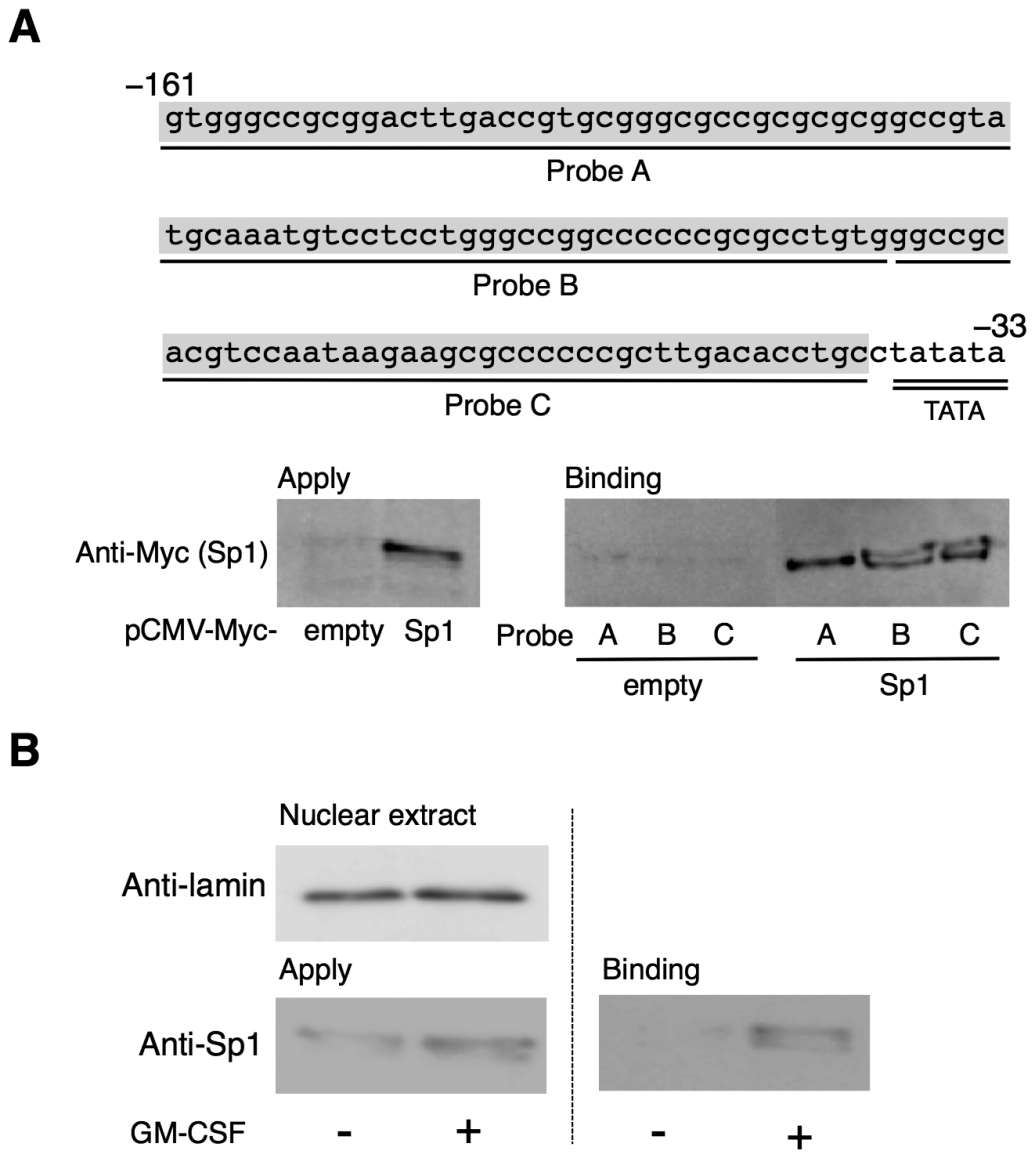
Sp1 binds to the *Aldh1a2* promoter region. (**A**) The locations and nucleotide sequences corresponding to Probe A, Probe B, and Probe C in the 5′-flanking region of the mouse *Aldh1a2* gene are shown. COS-7 cells were transfected with the 0.5 µg of pCMV-Myc-Sp1 or control empty vector. One day after transfection, cell lysates were analyzed for DNA-binding activity by DNAP assay using the indicated biotinylated DNA probes and anti-Myc Ab. **(B)** Flt3L-generated BM-DCs were cultured in the presence or absence of 10 ng/ml GM-CSF. After 16 h, nuclear extracts were analyzed for the presence of lamin B1 and Sp1 by Western blotting using anti-lamin B1 and anti-Sp1 Abs (*left panel*), or assessed for DNA binding activity by DNAP assay using biotinylated DNA Probe C and anti-Sp1 Ab (*right panel*). Data are representative of at least three independent experiments.

To examine whether Sp1 directly bound to the GC-rich region, we performed a DNAP assay with biotinylated DNA probes, A (−161 to −119), B (−118 to −82), and C (−81 to −40), which corresponded to the fragments of the 5′-flanking region of *Aldh1a2* (Figure 3A). Sp1 bound to all these probes, although it bound less efficiently to Probe A than to the other probes (Figure 3A). Because Probe C contained the essential region for Sp1-dependent *Aldh1a2* expression, we used Probe C to assess the DNA-binding capacity of Sp1 in the nuclei of BM-DCs.

Stimulating Flt3L-generated BM-DCs with GM-CSF enhanced the Sp1 protein levels in the nuclei of these cells and enhanced their binding capacity to Probe C (Figure 3B). Taken together, these results suggest that MAPK-dependent Sp1 nuclear translocation and its binding to the short GC-rich region near the TATA box are involved in GM-CSF-induced *Aldh1a2* expression in DCs.

### RAR and RXR participate in inducing *Aldh1a2* expression

**Figure 4:**
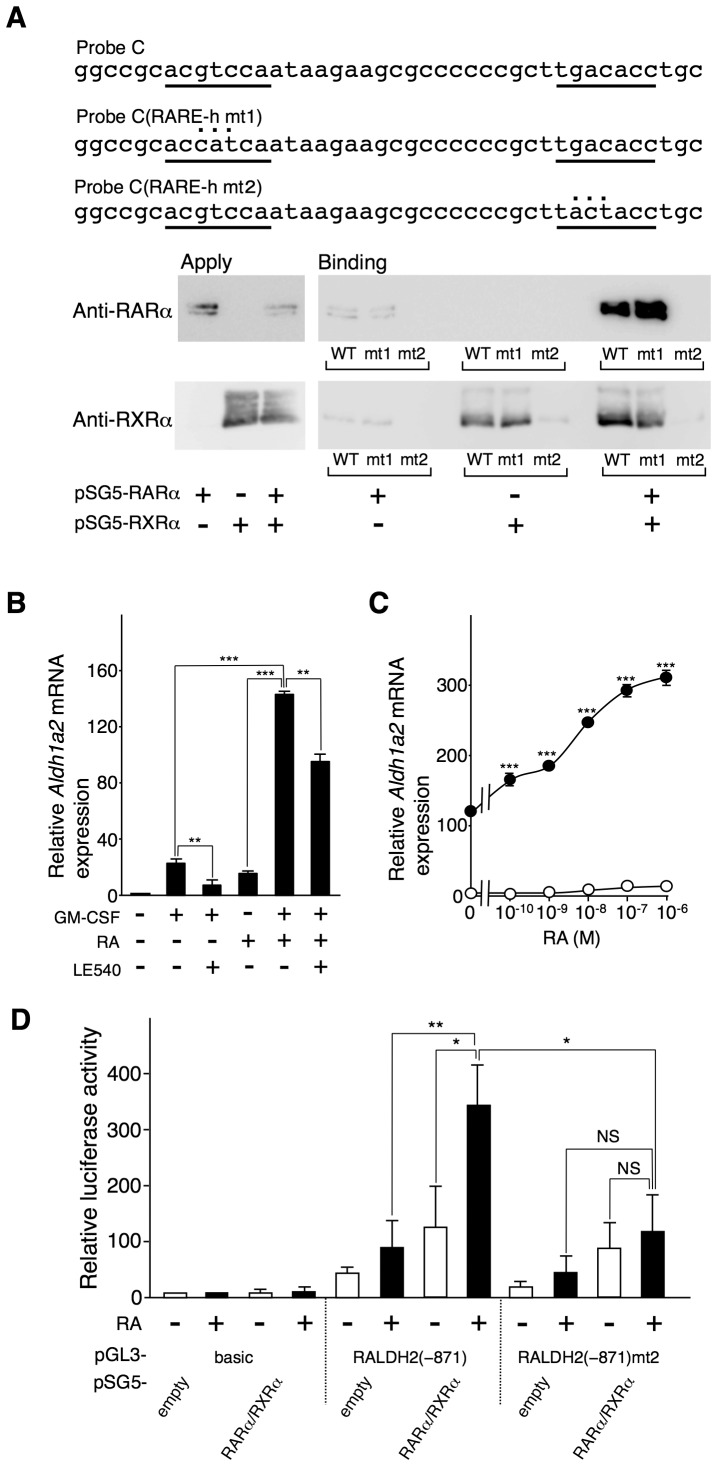
RA enhances GM-CSF-induced *Aldh1a2* expression via the RARα/RXRα heterodimer bound to the RARE half-site. (**A**) Localization of the putative RARE half-sites (*underlined*) in Probe C and their mutants, Probe C(RARE-h mt1) and Probe C(RARE-h mt2), are shown. COS-7 cells were transfected with the 0.5 µg of pSG5-RARα and/or pSG5-RXRα. One day after transfection, cell lysates were subjected to DNAP assay using the biotinylated DNA probes. The precipitates were analyzed by Western blotting using anti-RARα(*upper panel*) and RXRα (*lower panel*) Abs. (**B**) Flt3L-generated BM-DCs were cultured with 10 ng/ml GM-CSF for 16 h in the presence or absence of 100 nM RA. LE540 (1 µM) was added to the indicated cultures. After the culture, *Aldh1a2* mRNA expression was assessed by real-time PCR. The *Aldh1a2* mRNA expression level in the cells incubated with medium alone for 16 h was set to 1. (**C**) BM-DCs were cultured with (*closed circle*) or without (*open circle*) 10 ng/ml GM-CSF for 16 h in the presence of graded concentrations of RA. After the culture, *Aldh1a2* mRNA expression was assessed by real-time PCR. Relative expression levels were calculated by defining the *Aldh1a2* mRNA expression level in the cells incubated with medium alone for 16 h was set to 1. Asterisks indicate a significant difference (***p<0.001) from the *Aldh1a2* expression in BM-DCs stimulated with GM-CSF alone. (**D**) COS-7 cells were transfected in triplicate with 1.25 µg of the pGL3-RALDH2 (−873) reporter vector or that containing the mutated RARE half-site (mt2) with or without the 0.5 µg of expression vectors, pSG5-RARα and pSG5-RXRα. One day after transfection, cells were stimulated with or without 100 nM RA, and luciferase activities were measured. The relative promoter activities were calculated by arbitrarily defining the activity of pGL3-baic alone without RA as 1. Data in (B, C, and D) are presented as mean + SD (B and D) or mean ± SD (C) of triplicate cultures. Statistical significance between two groups was determined by the Student's *t* test (*p<0.05, **p<0.01, ***p<0.001; NS, not significant). Data are representative of at least three independent experiments.

Probe C contained the two putative RARE half-sites (−75 and −49). To test whether RARα or RXRα could bind to these putative RARE half-sites, we performed DNAP assays using biotinylated wild-type Probe C and its mutant DNA probes, Probe C(RARE-h mt1) and Probe C(RARE-h mt2); these mutant probes contained a mutation in the upstream putative RARE half-site and a mutation in the downstream putative RARE half-site, respectively (Figure 4A). RXRα alone but not RARα alone could bind to Probe C and Probe C(RARE-h mt1). RARα and RXRα mutually enhanced their binding to Probe C and Probe C(RARE-h mt1). RARα, RXRα, or their combination could not bind to Probe C(RARE-h mt2) (Figure 4A). These results suggest that RARα and RXRα can bind to the RARE half-site located adjacent to the TATA box.

RAR-mediated signaling appears to be essential for *Aldh1a2* expression in DCs. In vitamin A-deficient mice, *Aldh1a2* expression and RALDH2 activity in MLN-DCs and LP-DCs are markedly reduced [11], [17]–[19]. Accordingly, GM-CSF-induced *Aldh1a2* expression in BM-DCs was markedly suppressed by the RAR pan-antagonist LE540 (Figure 4B), as previously reported [11]. RA alone induced low *Aldh1a2* expression levels in Flt3L-generated BM-DCs; however, it significantly enhanced GM-CSF-dependent *Aldh1a2* expression in a dose-dependent manner (Figure 4C), in accordance with our previous observation [11]. Without the addition of any additional RA to the culture medium, retinol or a minute amount of RA in fetal bovine serum in the culture medium may have weakly stimulated RAR.

To assess the involvement of RARα and RXRα in the RA-dependent activation of *Aldh1a2* promoter activity, we transfected COS-7 cells with RARα and RXRαexpression vectors and a pGL3 reporter vector that contained a 1-kb fragment (−873 to +182) of the 5′-flanking region of *Aldh1a2* [pGL3-RALDH2 (−873)] (Figure 4D). *Aldh1a2* promoter activity was induced by the ectopic expression of RARα and RXRα and was markedly enhanced by RA stimulation. The mutation (mt2) in the RARE half-site significantly reduced this RA-induced promoter activity. These results suggest that RA induces *Aldh1a2* transcription through an RARα/RXRα complex bound to the RARE half-site adjacent between the critical Sp1-binding region and the TATA box.

### Sp1 and RARα/RXRα cooperatively contribute to RA-dependent *Aldh1a2* expression

**Figure 5:**
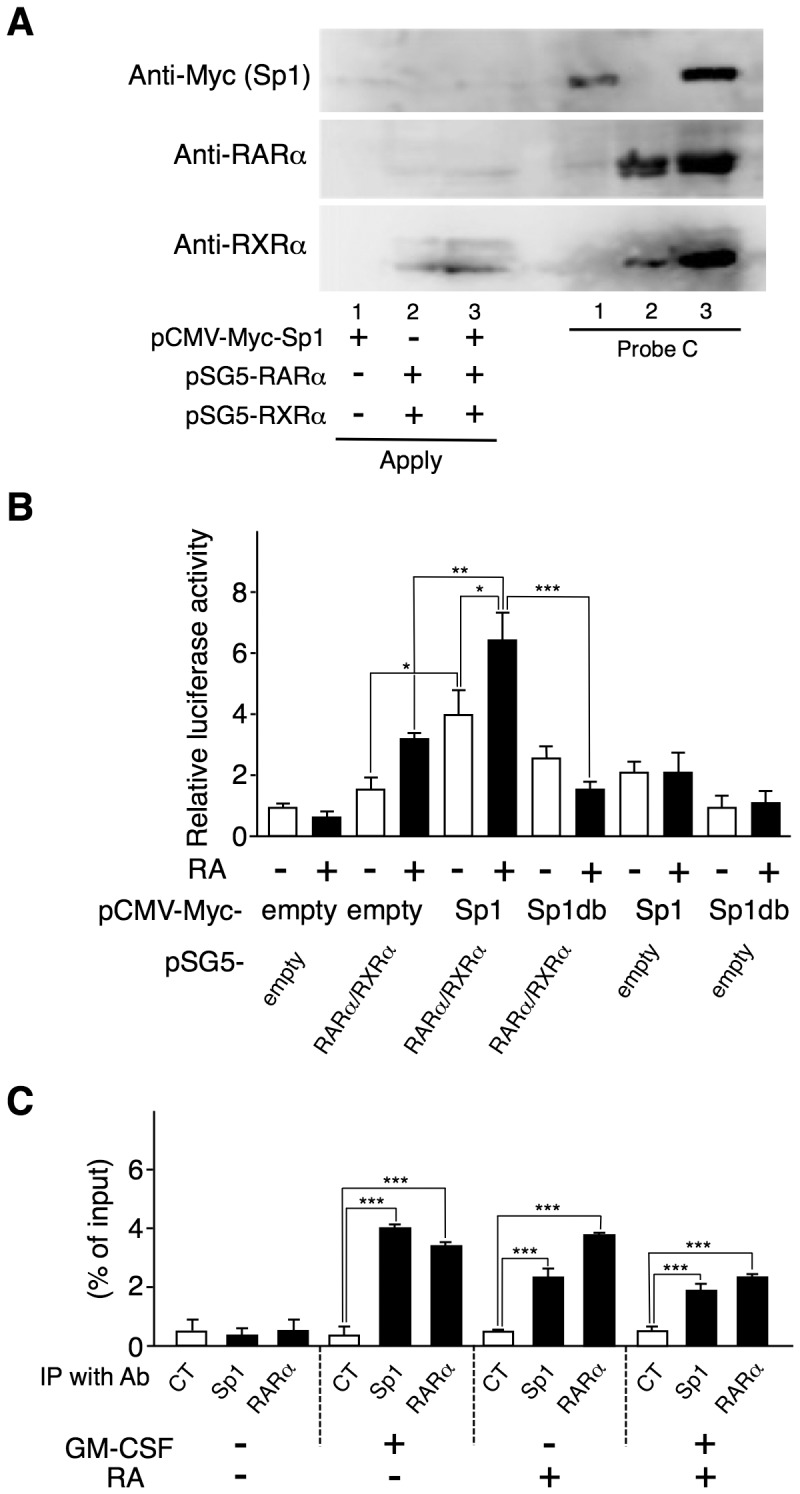
Sp1 and RARα/RXRα enhance each other's binding to the *Aldh1a2* promoter and cooperatively enhance its activity. (**A**) COS-7 cells were transfected with the 0.5 µg of pCMV-Myc-Sp1, the combination of pSG5-RARα and pSG5-RXRα, or the three. One day after transfection, cell lysates were subjected to DNAP assay using anti-Myc Ab, anti-RARα Ab, or anti-RXRα Ab, and biotinylated DNA Probe C whose sequence is shown in Figure 3. (**B**) COS-7 cells were transfected in triplicate with the 1.25 µg of pGL4-RALDH2 (−873) reporter vector and the 0.5 µg of expression vectors, pCMV-Myc-Sp1, pCMV-Myc-Sp1db, pSG5-RARα, and pSG5-RXRα, or control empty vectors. One day after transfection, cells were stimulated with or without 100 nM RA for 16 h. Then luciferase activities were measured. Relative promoter activities were calculated by arbitrarily defining the activity of pGL4-RALDH2 (−873) alone without RA as 1. (**C**) Flt3L-generated BM-DCs were cultured with or without 10 ng/ml GM-CSF or 10 nM RA. These cells were subjected to ChIP assay with anti-Sp1 or anti-RARα Ab or control IgG1. Binding of Sp1 and RARα proteins to the *Aldh1a2* promoter site was estimated by real-time PCR. Data in (B and C) are presented as mean + SD of triplicate cultures. Statistical significance between two groups was determined by the Student's *t* test (*p<0.05, **p<0.01, ***p<0.001). Data are representative of three independent experiments.

Sp1 can directly interact with RARα and RXRα [34], [35]. We examined whether Sp1 and RARα/RXRα mutually enhanced their binding to Probe C. As shown in Figure 5A, when Sp1 or the combination of RARα and RXRα was expressed in COS-7 cells, each of these could bind to this probe. However, when Sp1, RARα, and RXRα were expressed together, the binding of each component was significantly enhanced. These results suggest that Sp1 and RARα/RXRα mutually enhance their binding to the *Aldh1a2* promoter region. Accordingly, Sp1 and RARα/RXRα cooperatively enhanced *Aldh1a2* promoter-reporter activity in the presence of RA (Figure 5B). A truncated form of Sp1 that consisted only of the DNA-binding factor could not enhance the reporter activity in the presence or absence of RARα/RXRα (Figure 5B), and it suppressed Sp1-induced promoter activity in a dose-dependent manner (Figure S3). Mithramycin A and the MAPK pathway inhibitors PD98059 and SB204580 inhibited *Aldh1a2* expression that was induced by the combination of RA and GM-CSF (Figure S4A). These results suggest that Sp1 and RARα/RXRα cooperatively contribute to GM-CSF/RA-induced *Aldh1a2* expression, depending on MAPK activation.

Accordingly, ChIP assay results indicated that both RA and GM-CSF enhanced the binding of both Sp1 and RARα to this promoter region in BM-DCs, although no additional enhancement was found after stimulation with the combination of GM-CSF and RA in this assay (Figure 5C). The results suggest that adding either GM-CSF alone or RA alone more or less induces both Sp1 activation and RARα activation. Accordingly, mithramycin A, PD98059, and SB204580 also partially suppressed RA-induced *Aldh1a2* expression (Figure S4B).

**Figure 6:**
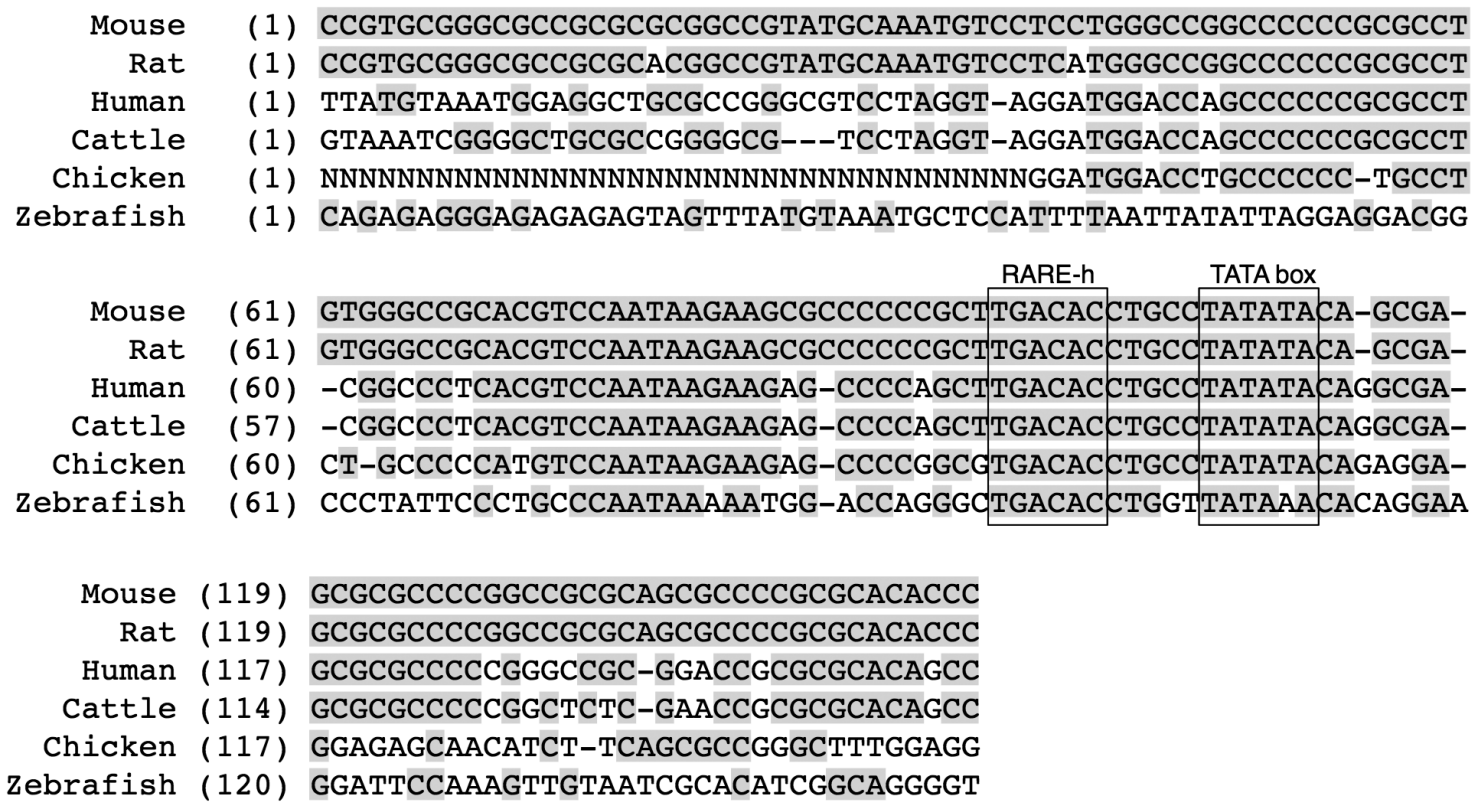
The short DNA regions containing the GC-rich region, the RARE half-site, and the TATA box in the 5′-flanking region of the *Aldh1a2* genes are well conserved among various species. The sequence of the 5′-flanking region of the mouse (*Mus musculus*) *Aldh1a2* gene was compared with that of the human (*Homo sapiens*) *ALDH1A2* gene, that of rat (*Rattus norvegicus*) *Aldh1a2* gene, that of cattle (*Bos taurus*) *ALDH1A2* gene, that of chicken (*Gallus gallus*) *ALDH1A2* gene, and that of zebrafish (*Danio rerio*) *aldh1a2* gene. The sequence data were obtained using the NCBI MapViewer. The shaded regions indicate homology with the mouse sequence. The locations of the conserved RARE half-sites (RARE-h) and TATA boxes are indicated by boxes.

Interestingly, the sequence of the RARE half-site adjacent to the TATA box of mouse *Aldh1a2* was identical to those of different species, including rat, human, cattle, chicken, and zebrafish (Figure 6). Furthermore, the sequences around the RARE half-sites in these species were highly conserved. These data suggest that the RARE half-site and the GC-rich Sp1-binding region are important for *Aldh1a2* expression.

### CpG methylation in the Aldh1a2 promoter region inhibits Sp1-dependent *Aldh1a2* promoter activation

**Figure 7:**
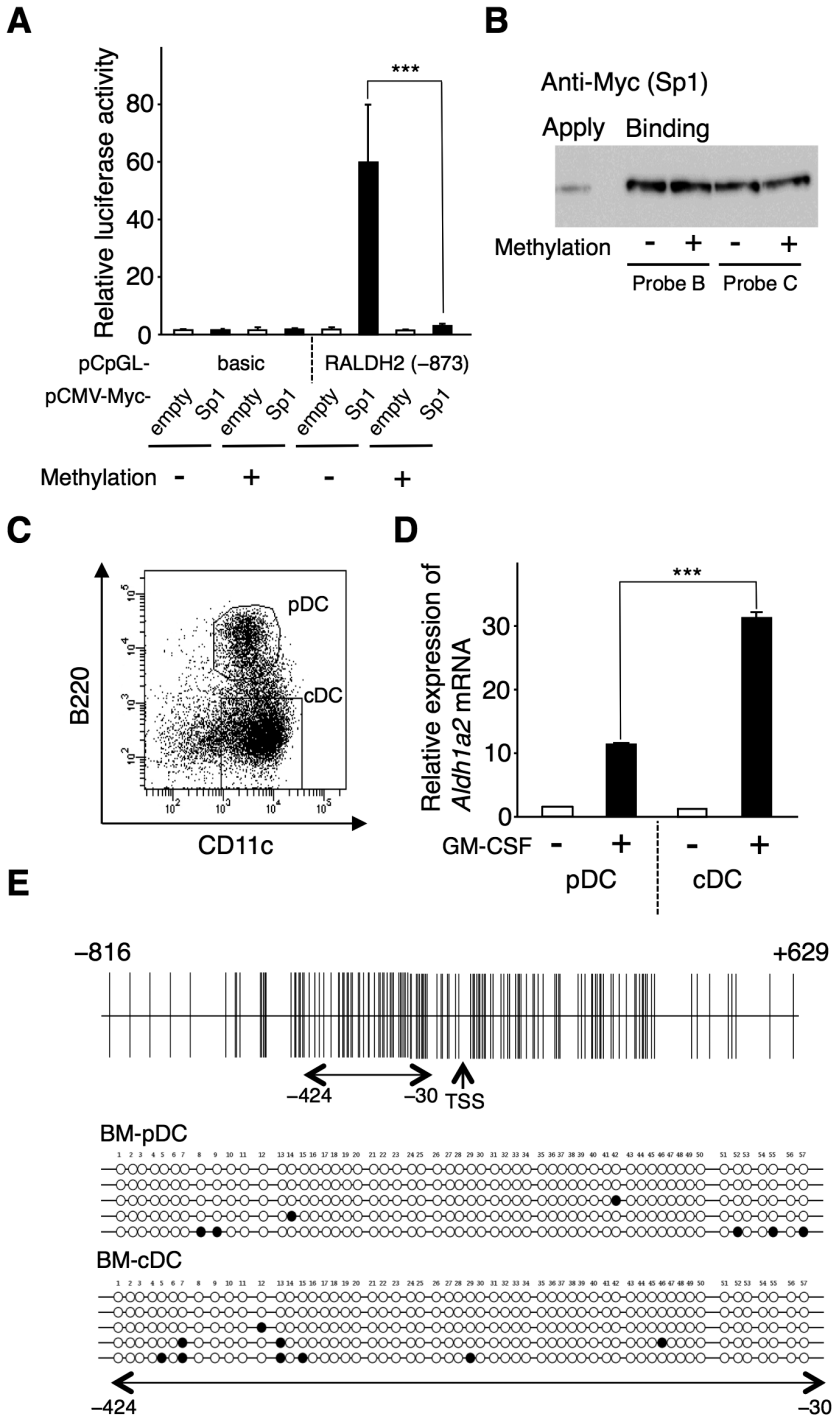
Methylation of the CpG island in the *Aldh1a2* promoter prohibits Sp1 to activate the promoter, whereas the *Aldh1a2* promoter is largely unmethylated in BM-pDCs as well as in BM-cDCs. (**A**) pCpGL-basic and pCpGL-RALDH2 (−873) reporter vectors were methylated with 1.25 µg of M.SssI. COS-7 cells were transfected with methylated or unmethylated pCpGL-basic or pCpGL-RALDH2 (−873) reporter vector in combination with or without the 0.5 µg of pCMV-Myc-Sp1 expression vector. One day after transfection, luciferase activity was measured. Relative promoter activities were calculated by arbitrarily defining the activity of pCpGL-basic alone as 1. (**B**) COS-7 cells were transfected with pCMV-Myc-Sp1. One day after transfection, cell lysates were analyzed for DNA binding activity by DNAP assay using DNA Probe B and Probe C methylated with M.SssI or left unmethylated. The bound proteins were analyzed by SDS-PAGE followed by Western blotting with anti-Myc Ab. (**C**) Flt3L-generated BM-DCs were stained with allophycocyanin-labeled anti-CD11c Ab and phycoerythrin-labeled anti-B220 Ab, and were sorted to cDC and pDC fractions with a FACSAria. (**D**) Sorted BM-pDCs and BM-cDCs were cultured for 16 h with or without 10 ng/ml GM-CSF. Expression of *Aldh1a2* mRNA was analyzed by real-time PCR. Relative expression levels were calculated by defining the *Aldh1a2* mRNA expression in the cells incubated with medium alone for 16 h was set to 1. Data in (A and D) are presented as mean + SD of triplicate cultures. Statistical significance between two groups was determined by the Student's *t* test (***p<0.001). Data in (A, B, and D) are representative of at least three independent experiments. (**E**) Genomic DNA was isolated from BM-pDCs and BM-cDCs, denatured, modified with sodium bisulfite, and used in nested PCR (−424 to −30) for bisulfite sequencing. Eleven and thirteen independent clones of pDCs and cDCs, respectively, were analyzed. The methylation patterns of 5 representative clones of each cell type are shown. Closed circles indicate methylated CpG and open circles indicate unmethylated CpG.

Because DNA methylation of CpG islands within proximal promoters is often associated with transcriptional regulation, we examined whether CpG methylation in the promoter region affected Sp1-dependent *Aldh1a2* transcription. The 5′-flanking region (−373 to +156) of *Aldh1a2* was inserted into a reporter vector that lacked CpG dinucleotides in its vector backbone (pCpGL) to avoid interference from methylation of the vector backbone. This reporter vector either remained unmethylated or was methylated in vitro using CpG methyltransferase (M.SssI). COS-7 cells were transfected with the methylated or unmethylated pCpGL-reporter vector together with the Sp1 expression vector (pCMV-Myc-Sp1) or an empty pCMV-Myc vector. Methylation of the *Aldh1a2* promoter region dramatically inhibited Sp1-dependent *Aldh1a2* promoter activity (Figure 7A), but did not affect Sp1 binding to the DNA oligonucleotides Probe B and Probe C derived from the *Aldh1a2* promoter region (Figure 7B). These results indicate that *Aldh1a2* transcription silencing by CpG methylation of the promoter region is not due to direct interference with Sp1 binding to the promoter region.

Flt3L-generated BM-DCs can be subdivided into two major subsets on the basis of their surface phenotypes: cDCs and pDCs. cDCs have been classically defined as B220^-^CD11c^hi^ DCs, whereas pDCs have been defined as B220^+^CD11c^int^ DCs (Figure 7C). In MLNs, *Aldh1a2* expression is found in cDCs; however, there is only minimal expression in pDCs [11], [36]. Accordingly, GM-CSF induced *Aldh1a2* expression in BM-cDCs but weakly induced this expression in BM-pDCs (Figure 7D).

We next examined whether methylation of the CpG sites in the promoter may control the responsiveness for *Aldh1a2* expression. A 395-bp region (−424 to −30) near TSS within the aforementioned 1,445-bp-long CpG island (Figure S1B) in BM-cDCs and BM-pDCs was assessed for the DNA methylation status by sodium bisulfite sequencing. We found that the CpG island was mostly unmethylated not only in cDCs but also in pDCs (Figure 7E). We also assessed the DNA methylation status of the 395-bp region in SPL-DCs cultured with or without GM-CSF. SPL-DCs express *Aldh1a2* after GM-CSF stimulation [11]. The CpG island was mostly unmethylated in SPL-DCs, with or without GM-CSF stimulation (Figure S5). Furthermore, the 395-bp region was also unmethylated in normal resident peritoneal macrophages and naïve CD4^+^ T cells (Figure S5), although these cells did not express *Aldh1a2* after GM-CSF stimulation in vitro (data not shown). These results indicate that in these normal immune cells, *Aldh1a2* expression is regulated by a DNA methylation-independent mechanism.

On the other hand, the 395-bp region in the murine macrophage-like cell line RAW264 and the murine DC-like cell line DC2.4 was hypermethylated (Figure S5). Both these cell lines did not express *Aldh1a2* after GM-CSF stimulation; however, after incubation with the DNA methylation inhibitor 5-aza-2′-deoxycytidine for 6 days, RAW264 cells began to express *Aldh1a2* after stimulation with GM-CSF or LPS (data not shown). These results suggest that DNA methylation of the GC-rich regions in the *Aldh1a2* promoter contributes to its unresponsiveness under certain circumstances.

## Discussion

The results of the present study indicated that the binding of Sp1 and RARα/RXRα to a GC-rich region and an RARE half-site, respectively, in a short 5′-flanking region of the TATA box was critical for the GM-CSF/RA-dependent induction of *Aldh1a2* expression. These short 5′-flanking regions were well conserved among different species, which suggested the importance of this region for initiating *Aldh1a2* transcription. DNAP and *Aldh1a2* promoter reporter assays using COS-7 cells indicated that Sp1 and the RARα/RXRα complex cooperatively enhanced their binding to this promoter and the reporter activity.

However, a ChIP assay using BM-DCs indicated that Sp1 and RARα binding was induced after adding either GM-CSF alone or RA alone. Because we found that at least residual RAR-mediated signaling appeared to be essential for GM-CSF-induced *Aldh1a2* expression in BM-DCs and that mithramycin A partially inhibited RA-induced *Aldh1a2* expression, it is likely that adding GM-CSF or RA more or less induced the activation of both Sp1 and RAR/RXR. Thus, even after adding GM-CSF or RA alone, Sp1 and RAR/RXR may also be able to cooperatively induce this promoter activation in BM-DCs. However, our ChIP assay showed that there was no additional coordinate effect when both GM-CSF and RA were added. Thus, it is possible that Sp1 and RARα bind to the *Aldh1a2* promoter transiently or coordinately allow another transcription factor to bind to this promoter. Because β-catenin-mediated signaling is essential for LP-DCs to express *Aldh1a1* and *Aldh1a2* mRNA [16], β-catenin may contribute to deliver an additional signal for *Aldh1a2* expression. Accordingly, GM-CSF has been suggested to stabilize the β-catenin protein, possibly by inactivating glycogen synthase kinase-3β during macrophage differentiation [37]. Nonetheless, any additional GM-CSF-dependent signal remains to be investigated.

GM-CSF and RA are involved in RALDH2 expression not only in mouse DCs but also in human DCs [38], [39]. In small intestine tissues and MLNs, GM-CSF is produced by various types of cells, including epithelial cells, Paneth cells, macrophages, and T cells, while RA may be produced by some types of cells including RALDH1^+^ intestinal epithelial cells, RALDH2^+^ cDCs, and *Aldh1a1* ^+^ *Aldh1a2* ^+^ *Aldh1a3* ^+^ MLN stromal cells [1], [19], [40]–[43]. Subsets of mature CD103^+^ cDCs in MLNs have high *Aldh1a2* expression levels and RALDH2 activity under steady-state conditions. A major population of RA-producing CD103^+^ MLN-DCs appears to be derived from the small intestinal LP [44], and most of CD103^+^ LP-DCs express weak RALDH2 activity [12]. Thus, the early conditioning of these DCs for RALDH2 expression may occur in the small intestinal LP or just after their arrival at MLNs. RA or an RAR agonist alone induces only low *Aldh1a2* expression levels in Flt3L-generated BM-DCs and SPL-DCs [11], [17]; however, we found that RA markedly enhanced GM-CSF-induced *Aldh1a2* expression in BM-DCs. Thus, RA is likely to contribute to this early conditioning.

It was reported that p38α deficiency or the oral administration of a MEK1/2-ERK inhibitor significantly inhibited *Aldh1a2* expression or RALDH2 activity in MLN-DCs [17], [30]. We found that the activation of ERK and p38 MAPK signaling pathways was also essential for GM-CSF-induced *Aldh1a2* expression and Sp1 translocation to the nuclei in BM-DCs. Immature DCs undergo a maturation process after stimulation with cytokines or TLR ligands via MAPK activation [45]–[48]. Accordingly, DC maturation is required for RALDH2 expression [11]. AU-rich elements (AREs) in the 3′-untranslated regions of cytokine genes are mRNA destabilizing elements [49]. p38 MAPK contributes to the expression of these cytokines by interfering with mRNA degradation through an ARE-targeted mechanism [50]. We also identified several AREs in the 3′-untranslated region of *Aldh1a2* (data not shown). Thus, p38 MAPK may also contribute to GM-CSF-induced *Aldh1a2* expression by stabilizing its mRNA through an AREs-dependent mechanism.

GM-CSF also activates the JAK2-STAT5 and STAT3 signaling pathways [31]. We found putative STAT binding sites in the 5′-flanking region of *Aldh1a2*. However, the expression of a constitutively active form of STAT5 was insufficient to directly activate or enhance *Aldh1a2* transcription (data not shown). Thus, STAT5 signaling may indirectly contribute to *Aldh1a2* expression by inducing an early response gene.

GM-CSF also activates NF-κB [31]. There are NF-κB-binding sites and a SREBP-binding site adjacent to the core promoter region of *Aldh1a2*, although we found that deleting these sites did not significantly reduce Sp1-dependent promoter activity. By up-regulating SREBP-1c, LXR ligands induce *Aldh1a1* and *Aldh1a2* expression in some tissues and cell lines [51], but fail to induce *Aldh1a2* expression in BM-DCs [11].

The GC-rich region in the *Aldh1a2* promoter was mostly unmethylated not only in Flt3L-generated BM-cDCs and SPL-DCs but also in BM-pDCs, macrophages, and naïve CD4^+^ T cells that did not have significant *Aldh1a2* expression. Thus, a DNA methylation-independent mechanism can also contribute to the regulation of *Aldh1a2* expression. pDCs express lower levels of GM-CSF receptors than cDCs in mice [52]. This may partly contribute to the differences in their *Aldh1a2* expression. Sp1 acetylation may also contribute to the regulation of *Aldh1a2* expression, as it has been reported that Sp1 acetylation is associated with loss of DNA binding at some promoters [53]. Accordingly, we found that histone deacetylase inhibitors inhibited GM-CSF-induced *Aldh1a2* expression in DCs (data not shown).

We revealed that Sp1- and RARα/RXRα-mediated signals were likely to be integrated through the *Aldh1a2* promoter covered by an unmethylated CpG island. Analyses of epigenetic modifications, such as histone acetylation, and possible involvement of additional transcription factors may clarify the mechanism underlying the selective regulation of *Aldh1a2* expression in specific DC subsets.

## Supporting Information

Figure S1 **Genomic organization of the mouse** ***Aldh1a2*** **gene and the distribution of CpG islands. (A)** The mouse *Aldh1a2* gene consists of 14 exons spanning more than 70 kb of genomic DNA. Six distinct CpG islands were identified using the NCBI MapViewer (http://www.ncbi.nlm.nih.gov/mapview/) analysis tool and are graphically represented here as gray blocks. (B) A 7,000-bp fragment (−3,355 to +3,645) of the mouse *Aldh1a2* gene, containing a transcription start site, was analyzed with the CpG island Searcher (http://www.uscnorris.com/cpgislands2). Filled bars represent the individual CpG residues. The CpG island (−816 to +629) around the promoter region is graphically represented here as gray blocks. The transcription start site (TSS) is indicated by a small arrow.
(TIFF)

Figure S2 **The** ***Aldh1a2*** **promoter activity is enhanced by the ectopic expression of Sp1 in a dose-dependent manner.** COS-7 cells were transfected in triplicate with the 1.25 µg of pGL3-RALDH2 (−2,600) reporter vector or control empty pGL3 basic vector in combination with graded concentrations of the pCMV-Myc-Sp1 expression vector and/or control empty vector, keeping the total dose of the latter two vectors constant at 2.5 µg. One day after transfection, luciferase activity was measured. Relative promoter activities were calculated by arbitrarily defining the activity of pGL3-basic alone as 1. Statistical significance between two groups was determined by the Student's *t* test (*p<0.05, **p<0.01; NS, not significant). Data are representative of three independent experiments.
(TIFF)

Figure S3 **The Sp1-induced** ***Aldh1a2*** **promoter activity is suppressed by the ectopic expression of a truncated form of Sp1 (Sp1db) that contains only of the DNA-binding domain in a dose-dependent manner.** COS-7 cells were transfected in triplicate with the 1.25 µg of pGL4-RALDH2 (−873) reporter vector, the pCMV-Myc-Sp1 expression vector or control empty vector, and pCMV-Myc-Sp1db expression vector, keeping the total dose of the latter three vectors constant at 2.5 µg. One day after transfection, cells were stimulated with or without 5 ng/ml PMA, and luciferase activity was measured. Relative promoter activities were calculated by arbitrarily defining the activity of pGL4-RALDH2 (−873) alone without PMA as 1. Statistical significance between two groups was determined by the Student's *t* test (**p<0.01, ***p<0.001).
(TIFF)

Figure S4 **Mithramycin A (MA), PD98059 (PD), and SB203580 (SB) inhibit GM-CSF/RA- or RA-induced** ***Aldh1a2*** **mRNA expression in BM-DCs.** Flt3L-generated BM-DCs were cultured with or without the combination of 10 ng/ml GM-CSF and 100 nM RA (A) or 100 nM RA (B) for 16 h in the presence or absence of 1 µM mithramycin A (MA), 50 µM PD98059 (PD), or 25 µM SB203580 (SB). After the culture, *Aldh1a2* gene expression was assessed by real-time PCR. The *Aldh1a2* mRNA expression level in the cells incubated with medium alone for 16 h was set to 1. Data are presented as mean + SD of triplicate cultures. Statistical significance between two groups was determined by the Student's *t* test (**p<0.01, ***p<0.001).
(TIFF)

Figure S5 **Methylation statuses of the CpG island in the** ***Aldh1a2*** **promoter region in SPL-DCs, macrophages, naïve CD4^+^ T cells, DC2.4, and RAW264 cells.** Bisulfite-PCR amplified using upstream *Aldh1a2* promoter-specific primers. SPL-DCs were cultured with or without 10 ng/ml GM-CSF for 24 h. Macrophages and naïve CD4^+^ T cells were isolated as described in Materials and Methods. Genomic DNA was isolated from the indicated cells, denatured, modified with sodium bisulfite, and used in nested PCR (−424 to −30) for bisulfite sequencing. Seven SPL-DC (−GM-CSF), 6 SPL-DC (+GM-CSF), 14 macrophage, 9 naïve CD4^+^ T cell, 16 DC2.4, and 23 RAW264 independent clones were analyzed. The methylation patterns of 5 representative clones of each cell type are shown. Closed circles indicated methylated CpG and open circles indicate unmethylated CpG.
(TIFF)

Table S1 **Sequences of the primers used.**
(PDF)
