# Supplementary figures and images for: Retinoic Acid and GM-CSF Coordinately Induce Retinal Dehydrogenase 2 (RALDH2) Expression through Cooperation between the RAR/RXR Complex and Sp1 in Dendritic Cells
Source: PLoS One. 2014 May 2;9(5):e96512. doi: 10.1371/journal.pone.0096512 (PMC4008585; doi:10.1371/journal.pone.0096512)

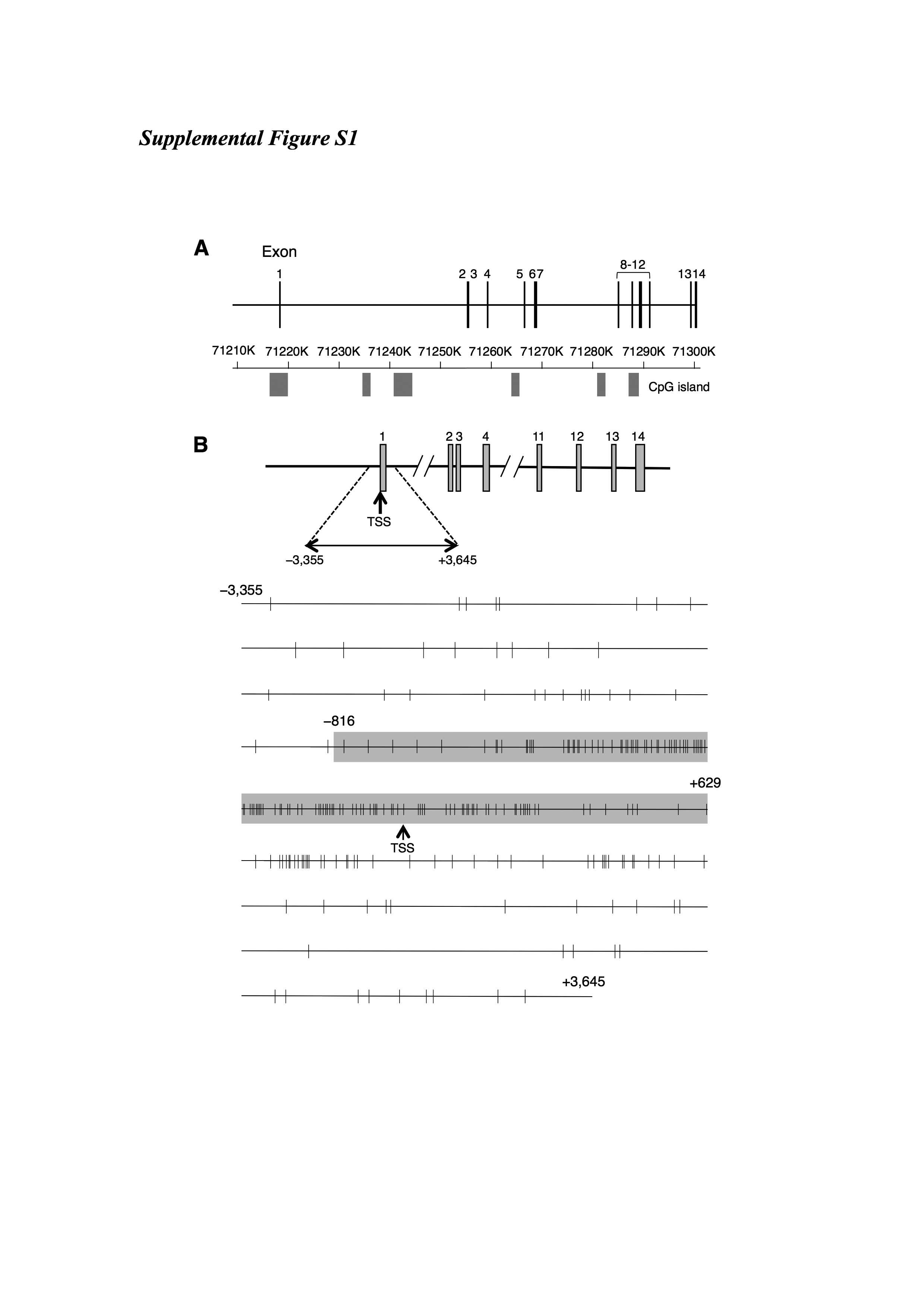

Supplement: Figure S1 — Genomic organization of the mouse Aldh1a2 gene and the distribution of CpG islands. (A) The mouse Aldh1a2 gene consists of 14 exons spanning more than 70 kb of genomic DNA. Six distinct CpG islands were identified using the NCBI MapViewer (http://www.ncbi.nlm.nih.gov/mapview/) analysis tool and are graphically represented here as gray blocks. (B) A 7,000-bp fragment (−3,355 to +3,645) of the mouse Aldh1a2 gene, containing a transcription start site, was analyzed with the CpG island Searcher (http://www.uscnorris.com/cpgislands2). Filled bars represent the individual CpG residues. The CpG island (−816 to +629) around the promoter region is graphically represented here as gray blocks. The transcription start site (TSS) is indicated by a small arrow. (TIFF) [file pone.0096512.s001.tif]

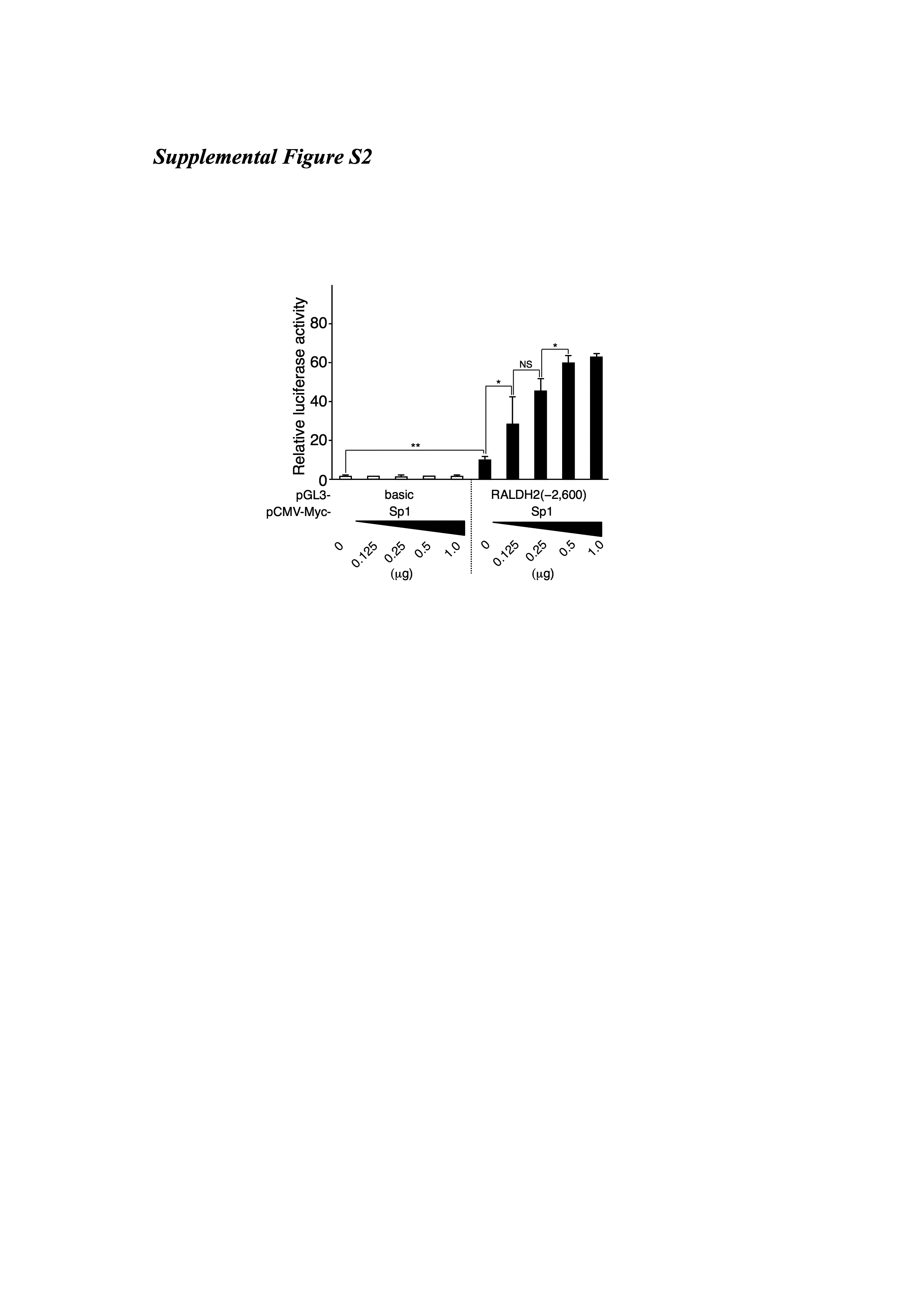

Supplement: Figure S2 — The Aldh1a2 promoter activity is enhanced by the ectopic expression of Sp1 in a dose-dependent manner. COS-7 cells were transfected in triplicate with the 1.25 µg of pGL3-RALDH2 (−2,600) reporter vector or control empty pGL3 basic vector in combination with graded concentrations of the pCMV-Myc-Sp1 expression vector and/or control empty vector, keeping the total dose of the latter two vectors constant at 2.5 µg. One day after transfection, luciferase activity was measured. Relative promoter activities were calculated by arbitrarily defining the activity of pGL3-basic alone as 1. Statistical significance between two groups was determined by the Student's t test (*p<0.05, **p<0.01; NS, not significant). Data are representative of three independent experiments. (TIFF) [file pone.0096512.s002.tif]

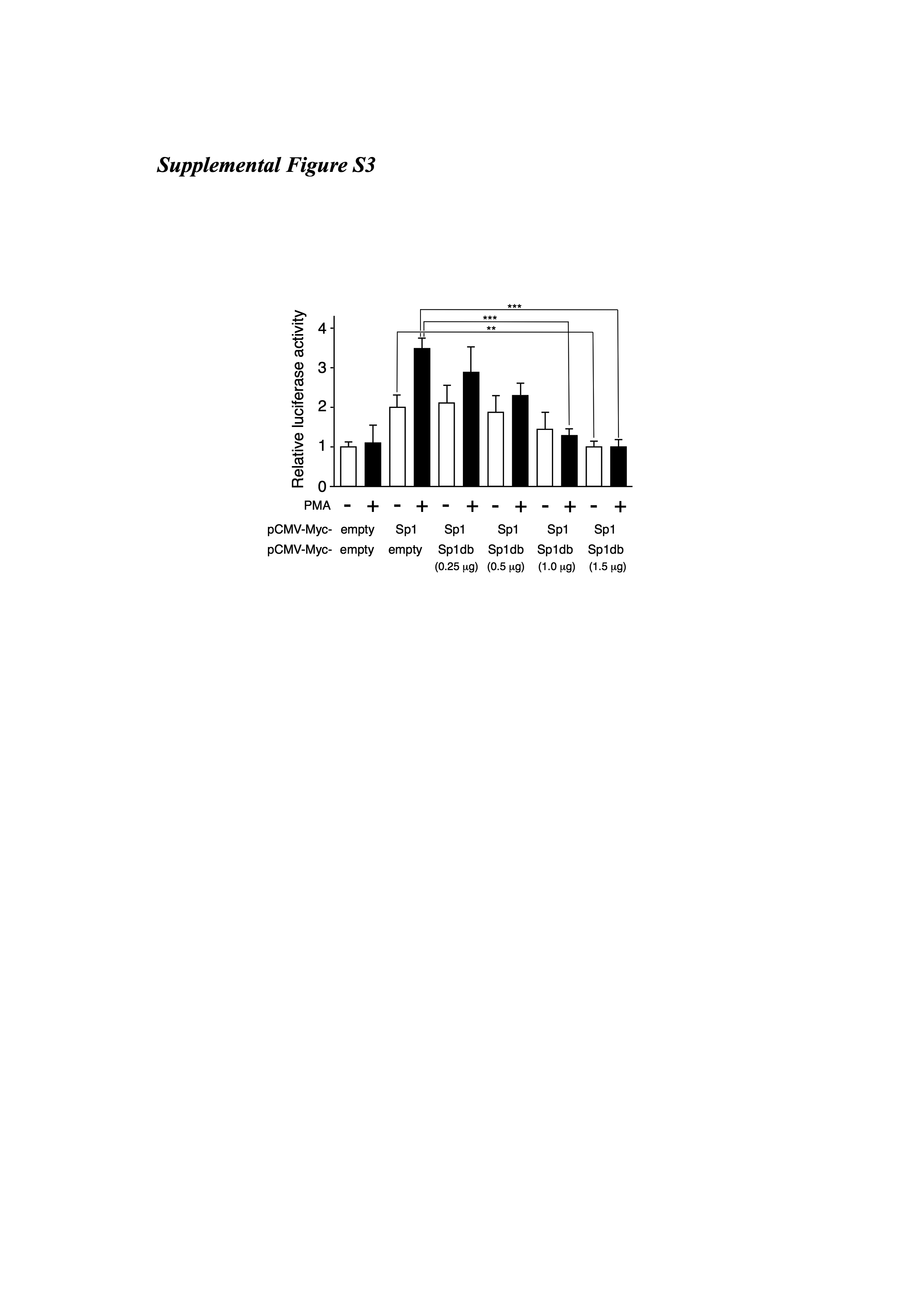

Supplement: Figure S3 — The Sp1-induced Aldh1a2 promoter activity is suppressed by the ectopic expression of a truncated form of Sp1 (Sp1db) that contains only of the DNA-binding domain in a dose-dependent manner. COS-7 cells were transfected in triplicate with the 1.25 µg of pGL4-RALDH2 (−873) reporter vector, the pCMV-Myc-Sp1 expression vector or control empty vector, and pCMV-Myc-Sp1db expression vector, keeping the total dose of the latter three vectors constant at 2.5 µg. One day after transfection, cells were stimulated with or without 5 ng/ml PMA, and luciferase activity was measured. Relative promoter activities were calculated by arbitrarily defining the activity of pGL4-RALDH2 (−873) alone without PMA as 1. Statistical significance between two groups was determined by the Student's t test (**p<0.01, ***p<0.001). (TIFF) [file pone.0096512.s003.tif]

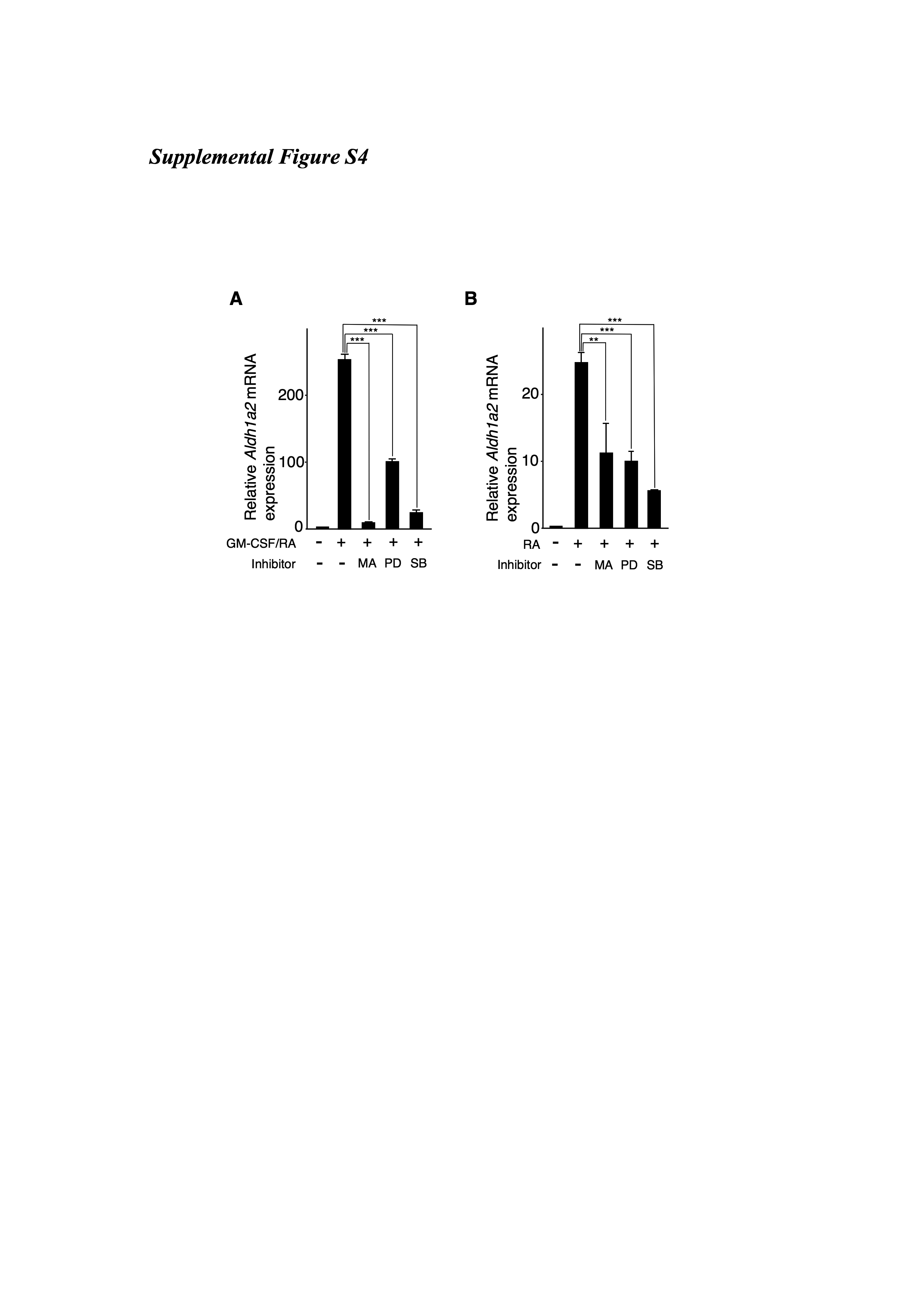

Supplement: Figure S4 — Mithramycin A (MA), PD98059 (PD), and SB203580 (SB) inhibit GM-CSF/RA- or RA-induced Aldh1a2 mRNA expression in BM-DCs. Flt3L-generated BM-DCs were cultured with or without the combination of 10 ng/ml GM-CSF and 100 nM RA (A) or 100 nM RA (B) for 16 h in the presence or absence of 1 µM mithramycin A (MA), 50 µM PD98059 (PD), or 25 µM SB203580 (SB). After the culture, Aldh1a2 gene expression was assessed by real-time PCR. The Aldh1a2 mRNA expression level in the cells incubated with medium alone for 16 h was set to 1. Data are presented as mean + SD of triplicate cultures. Statistical significance between two groups was determined by the Student's t test (**p<0.01, ***p<0.001). (TIFF) [file pone.0096512.s004.tif]

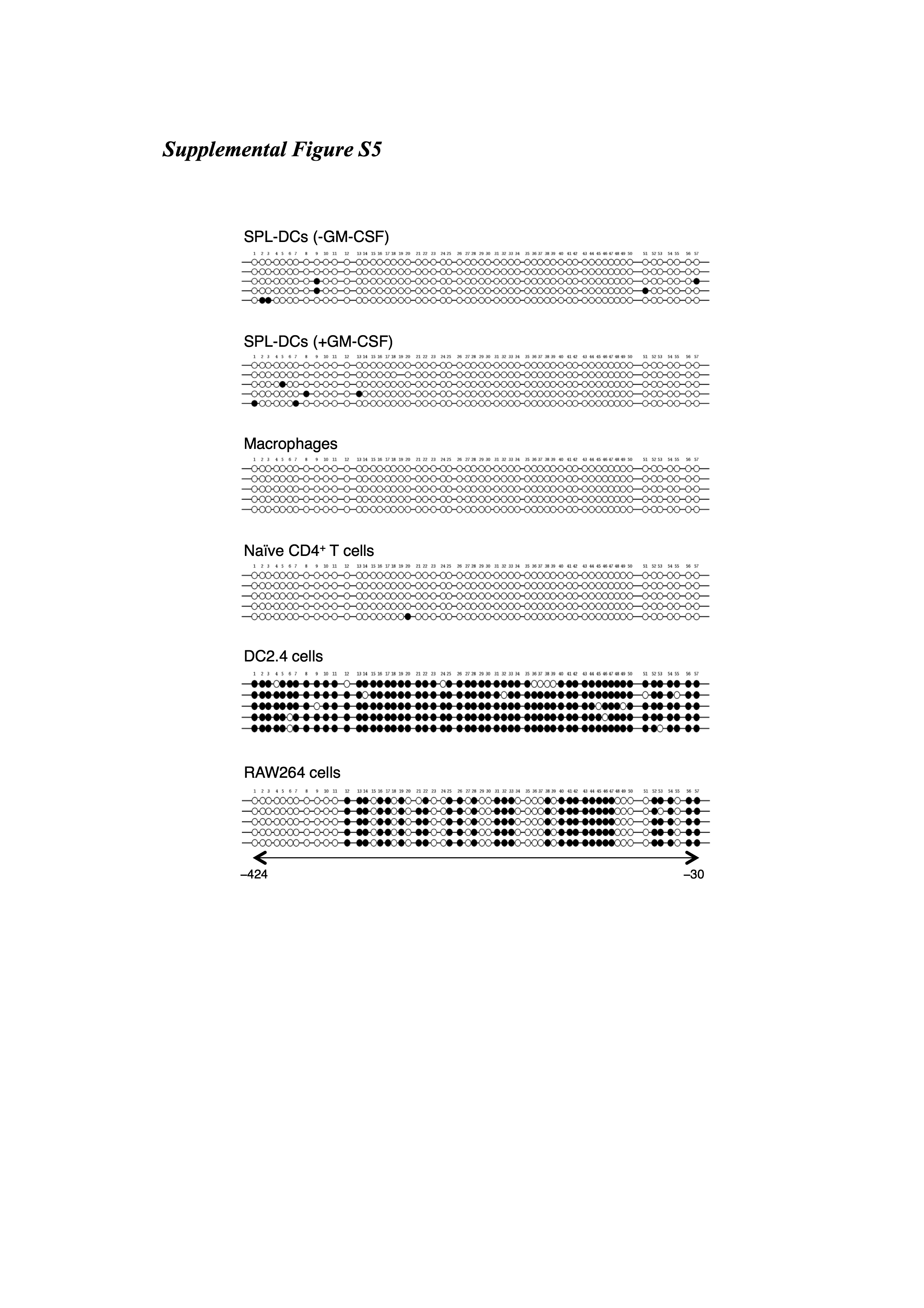

Supplement: Figure S5 — Methylation statuses of the CpG island in the Aldh1a2 promoter region in SPL-DCs, macrophages, naïve CD4+ T cells, DC2.4, and RAW264 cells. Bisulfite-PCR amplified using upstream Aldh1a2 promoter-specific primers. SPL-DCs were cultured with or without 10 ng/ml GM-CSF for 24 h. Macrophages and naïve CD4+ T cells were isolated as described in Materials and Methods. Genomic DNA was isolated from the indicated cells, denatured, modified with sodium bisulfite, and used in nested PCR (−424 to −30) for bisulfite sequencing. Seven SPL-DC (−GM-CSF), 6 SPL-DC (+GM-CSF), 14 macrophage, 9 naïve CD4+ T cell, 16 DC2.4, and 23 RAW264 independent clones were analyzed. The methylation patterns of 5 representative clones of each cell type are shown. Closed circles indicated methylated CpG and open circles indicate unmethylated CpG. (TIFF) [file pone.0096512.s005.tif]
