# Supplementary material for: Retinoic Acid and GM-CSF Coordinately Induce Retinal Dehydrogenase 2 (RALDH2) Expression through Cooperation between the RAR/RXR Complex and Sp1 in Dendritic Cells
Source: PLoS One. 2014 May 2;9(5):e96512. doi: 10.1371/journal.pone.0096512 (PMC4008585; doi:10.1371/journal.pone.0096512)
Supplement: Table S1 — Sequences of the primers used. (PDF) [file pone.0096512.s006.pdf]

Table S1. **Sequences of the primers used.**

**For real-time PCR**	Primer Sequence (5'-3')	
Aldh1a2	Forward	TGCCATGTTCATCTCCAACTG
	Reverse	AGAAACGTGGCAGTCTTGGC
Rplp0	Forward	GGTGCCACACTCCATCATCA
	Reverse	CGCAAATGCAGATGGATCAG
**For bisulfite sequencing**		
1st PCR	Forward	ATTTGGAATATTTAGGTAATTT
	Reverse	CATATATATAAACAAATATCAAA
nested PCR	Forward	GAGTATTTATTATTTTATTTAG
	Reverse	CATATATATAAACAAATATCAAA
**For reporter vector construction**		
pGL3-RALDH2 (-2,600)	Forward	TAGGTACCGTTGTTTTTGGACCCGAGGA
pGL3-RALDH2 (-873)	Forward	ATGGTACCGGCTGTGGGATTTTCA
pGL3-RALDH2 (-373)	Forward	ATGGTACCGGCTGAGCGAAAGCTCTT
pGL3-RALDH2 (-154)	Forward	ATGGTACCGCGGACTTGACCGTGCGG
pGL3-RALDH2 (+81)	Forward	ATGGTACCATGGCCTCGCTGCTGCAGCTC
	Reverse	GCAAGCTTTCCAGAGGATGCTTCCAGAAA
**For expression vector construction**		
pCMV-Myc-Sp1	Forward	GCGAATTCATGAGCGACCAAGATCACTC
	Reverse	GCGTCGACTTAGAAACCATTGCCACTGA
pCMV-Myc-Sp1db	Forward	GCGAATTCTCAGGAGATCCTGGCAAA
	Reverse	GCGTCGACTTAGAAACCATTGCCACTGA
**For mutagenesis**		
pGL3-RALDH2 (-873) mt2	Forward	*CCAATAAGAAGCGCCCCCCGCTTactACCT GCCTATATACAGCGAGCGCG
	Reverse	*CGCGCTCGCTGTATATAGGCAGGTagtAAG CGGGGGGCGCTTCTTATTGG
pGL3-RALDH2(-373)Δ1	Forward	CGCTTCTTATTGGACGTGCG
	Reverse	GCCTATATACAGCGAGCGCG
pGL3-RALDH2(-373)Δ2	Forward	CCAGGAGGACATTTGCATAC
	Reverse	CGCACGTCCAATAAGAAGCG

pGL3-RALDH2(-373)Δ3	Forward	CACGGTCAAGTCCGCGGCCCAC
	Reverse	GTATGCAAATGTCCTCCTGG
**For ChIP**		
	Forward	CCGTATGCAAATGTCCTCCTGG
	Reverse	CGATCTCGCTGGAAGTCATG

*The lower-case letters indicate the mutated sequences.
